# Factors Related to Suicide Attempts: The Roles of Childhood Abuse and Spirituality

**DOI:** 10.3389/fpsyt.2021.565358

**Published:** 2021-03-29

**Authors:** Hyejin Tae, Jeong-Ho Chae

**Affiliations:** ^1^Stress Clinic, Health Promotion Center, Seoul St. Mary's Hospital, The Catholic University of Korea, Seoul, South Korea; ^2^Department of Psychiatry, Seoul St. Mary's Hospital, The Catholic University of Korea, Seoul, South Korea

**Keywords:** suicide attempts, depression, childhood sexual abuse, childhood emotional abuse, spirituality

## Abstract

**Objectives:** The purpose of this article was to identify independent factors associated with suicide attempts in patients with depression and/or anxiety.

**Background and Aims:** This study was conducted in order to examine whether risk and protective psychological factors influence the risk of suicide attempts among outpatients with anxiety and/or depressive disorders. In this regard, explanatory models have been reported to detect high-risk groups for suicide attempt. We also examined whether identified factors serve as mediators on suicide attempts.

**Materials and Methods:** Patients from 18 to 65 years old from an outpatient clinic at Seoul St. Mary's Hospital were invited to join clinical studies. From September 2010 to November 2017, a total of 737 participants were included in the final sample. The Beck Depression Inventory (BDI), State-Trait Anxiety Inventory (STAI), Childhood Trauma Questionnaire (CTQ), Functional Assessment of Chronic Illness Therapy-Spiritual Well-being Scale (FACIT-Sp-12), and Functional Social Support Questionnaire (FSSQ) were used to assess psychiatric symptoms. An independent samples *t*-test, a chi-square test, hierarchical multiple regression analyses, and the Baron and Kenny's procedures were performed in order to analyze data.

**Results:** Young age, childhood history of emotional and sexual abuse, depression, and a low level of spirituality were significant independent factors for increased suicide attempts. Depression was reported to mediate the relationship between childhood emotional and sexual abuse, spirituality, and suicide attempts.

**Conclusions:** Identifying the factors that significantly affect suicidality may be important for establishing effective plans of suicide prevention. Strategic assessments and interventions aimed at decreasing depression and supporting spirituality may be valuable for suicide prevention.

## Introduction

Approximately 90% of people who commit suicide are considered to have at least one psychiatric disorder at the time of death (1, 2). Of these psychiatric disorders, mood and anxiety disorders have been particularly identified as critical determinants of suicide (3–6). In addition, sociodemographic factors, such as being male, unmarried, and unemployed (7), as well as exposure to childhood trauma (8) are recognized as independent risk factors for suicide. Earlier studies have showed that childhood abuse is an important risk factor for violent behaviors toward the self and a key factor to consider for the effective prevention of suicide (9–11). Several studies have also suggested that childhood abuse is related to internalizing (depression and anxiety) and externalizing (substance abuse and antisocial behavior) dimensions underlying psychiatric disorders, and that both of these dimensions are related to suicide attempts (12, 13).

Although there is a substantial amount of literature on suicide risk factors (14, 15), much less is known about the protective factors for suicide (16). In recent decades, some researchers have started to recognize the importance of identifying these protective factors (17, 18). Several studies have reported that spirituality is associated with lower suicide risk and better mental health (19, 20). Spirituality has been defined as “the personal search for understanding life's meaning and the goal of life” (21). Koenig et al. (21) reported that spiritual belief and practices such as meditation, prayer, and communal worship tend to arouse positive and supportive emotions from participants. Other studies have also indicated that the protective effect of spiritual values might be affected by the influence of social support (22). The presence of social support may increase feelings of belongingness, which are negatively associated with suicide risk within Joiner's Interpersonal Theory of Suicide (23, 24). Given the importance of mental illness in the context of suicide (25), it is important to understand the relationship between spirituality, social support, and suicide attempts in populations of people with depression.

Regardless of the agreement among the empirical studies on whether the presence of spiritual beliefs and social support is related to increased resiliency to suicide, the majority of previous studies have not considered both spirituality and social support for suicide. Therefore, studies which examine both factors affecting suicide would be meaningful for suicide protection. In particular, spirituality has some characteristics such as social practices similar to social support. To differentiate both positive factors, we considered spirituality as spiritual beliefs, an affective aspect of spirituality and a cognitive aspect of spirituality. The spiritual practices including communal worship were excluded to differentiate the other positive factor such as social support. Therefore, we identified both relationships between spirituality and suicide attempts, and between social support and suicide attempts, respectively.

The purpose of this study was to examine whether risk and protective factors influence the risk of suicide attempts among outpatients with anxiety and/or depressive disorders. First, we classified patients with anxiety and/or depressive disorder into two groups by the presence or absence of a history of suicide attempts. Then, both groups were compared in terms of sociodemographic factors, psychiatric symptoms, histories of childhood trauma, and positive psychological factors such as spirituality and social support. We used suicide attempts as an outcome variable because suicide attempts are a definite indicator of suicidality (26). We then evaluated the effects of the positive and negative factors which were considered to be related to suicide attempts. Finally, we examined whether the identified significant factors served as mediators on the risk of suicide attempts. According to the prior research findings described above, we formulated three hypotheses: that childhood abuse among different types of childhood maltreatment would be related to the increased suicide attempts, that positive factors such as spirituality and social support would be related to the decreased suicidal behavior, and that depression would mediate the relationship between significant positive/negative factors and suicide attempts.

## Methods

### Participants and Procedures

Treatment-seeking patients from 18 to 65 years old from the Mood and Anxiety Disorders Clinic at Seoul St. Mary's Hospital, The Catholic University of Korea were invited to join clinical studies. From September 2010 to November 2017, the participants underwent an assessment that comprised several health and behavioral aspects as well as a psychological evaluation at their first visit before treatment. Patients who met the DSM-IV criteria for depressive and/or anxiety disorders were included in this study. Relevant diagnoses were made by a psychiatrist using structured interviews of the Mini-International Neuropsychiatric Interview (M.I.N.I) (27). A lifetime diagnosis of psychotic disorder, bipolar disorder, intellectual disability, current substance abuse, any mental disorder due to another medical condition as well as medical problems affecting study participation were exclusion criteria. A total of 751 outpatients who met the inclusion criteria participated in the study and were administered a battery of self-report psychiatric questionnaires. Excepting analyses of those who had not completed all measures, the final sample included 737 patients. This investigation was carried out in accordance with the latest version of the Declaration of Helsinki. This study was approved by the Institutional Review Board of the Ethics Committee of Seoul St. Mary's Hospital at The Catholic University of Korea (KC09FZZZ0211). Written informed consent was obtained from all subjects after providing them with a complete description of the study.

### Measurements

#### Sociodemographic, Clinical Information, and Suicide Attempts

Sociodemographic and clinical information were acquired from medical charts and interviews with patients and their caretakers. Data on age, gender, education, marriage status and employment status were obtained in the study. Education was estimated by the years of formal education. Marital status was categorized as married and unmarried (including single, divorced, and widowed). Employment status was categorized as employed (including permanent and precarious employment) and unemployed. In this study, the definition of unemployed included all subjects who were without work regardless of whether they were looking for work or not. Suicidality was assessed according to the following question: “Have you ever attempted suicide in the past?” There is supporting evidence that past suicide attempt is a leading risk factor for future attempts and death by suicide (28, 29). Positive responses were confirmed by follow-up questions that assessed the number, method, and subjective seriousness of these attempts.

#### Psychiatric Symptoms

The Beck Depression Inventory (BDI) was used to assess depressive symptoms. The BDI consists of 21 items on a 4-point scale from 0 (symptom absent) to 3 (severe symptoms) and is a self-report inventory for evaluating the severity of depression. The BDI evaluates depressive symptoms within the preceding week, with high scores reflecting a greater severity of depressed mood (range = 0–63) (30). The Korean version of BDI used here has been validated (31–33).

Anxiety was evaluated by the State-Trait Anxiety Inventory (STAI). The STAI consists of two scales, each containing 20 items using a 4-point Likert scale. First, the State Anxiety Scale (S-Anxiety) assesses the current state of anxiety, asking how respondents feel “right now,” including subjective feelings of apprehension, tension, nervousness, worry, and activation/arousal of the autonomic nervous system. The Trait Anxiety Scale (T-Anxiety) assesses relatively stable aspects of “anxiety proneness” using items that measure general states of calmness, confidence, and security. When the scores on each item are added up, a total summed score is obtained. We used a total summed score to assess anxiety. The range of scores for each subtest is 20–80, with a higher score implying greater anxiety (34). Validation of the Korean version of STAI has proven its reliability and sensitivity in the measurement of anxiety (35).

#### Childhood Trauma

Childhood trauma was measured using the short form of the Childhood Trauma Questionnaire (CTQ). The CTQ yields scores for childhood physical neglect, emotional neglect, physical abuse, emotional abuse, and sexual abuse, as well as a total score. The short version of CTQ is a 5-item Likert scale on which the respondents rate the frequency of 28 sentences about childhood trauma experiences (36–38). The CTQ measures the severity of symptoms as none, low, moderate, or severe, and we used a moderate-to-severe cutoff point (39). Validation of the Korean version of CTQ has proven its reliability and sensitivity (40).

#### Positive Psychological Factors

Spiritual well-being was assessed using the Functional Assessment of Chronic Illness Therapy-Spiritual Well-being Scale (FACIT-Sp-12) (41). This scale is divided into three factors: faith (spiritual beliefs), peace (an affective aspect of spirituality), and meaning (a cognitive aspect of spirituality) (42, 43). The FACIT-Sp-12 has a 5-point Likert-style response scale (0 = not at all, 1 = a little bit, 2 = somewhat, 3 = quite a bit, 4 = very much), and when the scores on all the items are added up, a total summed score is obtained (two items are reverse-scored). The Korean version has not been officially validated, but it has been used in a domestic study. In that study, Cronbach alpha was 0.751, and spiritual well-being correlated negatively with anxiety (r = −0.613) and depression (r = −0.526, all *p* < 0.05), attesting to the concurrent validity of the FACIT-sp (44). The internal consistency of our study as measured by the Cronbach's alpha coefficient was been found to be 0.76 for the Spiritual Well-being subscale.

Social support was measured by the Functional Social Support Questionnaire (FSSQ), an eight-item questionnaire which assesses the strength of one's social network. It consists of two domains: confidential social support and affective social support. The score of the scale ranges from 11 to 55, where 55 indicates the highest levels of social support. Validation of the Korean version of FSSQ has proven its reliability and sensitivity in the measurement of social support (45).

### Data Analysis

Participants were classified into either the suicide attempt group or the no suicide attempt group based on the responses of a self-report form assessing suicidality. The characteristics of the participants were reported as means (standard deviation [SD]) for continuous variables, and as numbers (%) for categorical variables. Two-tailed tests were used in all instances, and statistical significance was defined as *p* < 0.05 with confidence intervals at 95%. In order to identify the relationship between each of these variables and suicide attempts, an independent samples *t*-test was performed for each continuous variable and a chi-square test was performed for each categorical variable.

Hierarchical multiple regression analyses were performed in order to evaluate whether each positive and negative factor is related to suicide attempts or not. The principle of “factors coming earlier in the series can affect other factors coming later, but not vice versa” was used to decide the order of the included variables (46). In the four-block model, we initially included demographic data to adjust for the effects of demographic differences. Then, childhood trauma, positive psychological factors, and clinical symptoms were entered into blocks 2, 3, and 4, respectively, according to the guidelines. Because childhood trauma is known as one of the distal factors which affect suicide risk (4), we entered this variable into block 2. Next, positive factors such as spirituality and social support have long been regarded as mitigating distress (47, 48). Social support has been found to be inversely related to depression and anxiety (49). Moreover, spirituality plays a key role in helping depressive patients cope with stress (50, 51). Because protective factors have been known to decrease psychiatric symptoms such as depression and anxiety, positive factors and clinical symptoms were included in blocks 3 and 4. A forward selection method was used in the multiple regression analyses because intercorrelations between variables were expected.

In the final part of the study, we performed mediating analysis in order to understand the relationship between significant factors and suicidal ideation. Baron and Kenny's procedures were used to examine the mediating effect of depression (52). Past studies have shown that the association between childhood abuse and suicidal behaviors was mediated by mental disorders such as depression (53–55). In addition, other studies have described that spirituality and social support provide protective effects regarding suicide attempts (56) and are associated with decreased rates of depression (57). According to these previous studies, we confirmed a relationship between each independent variable (i.e., childhood trauma and positive factors) and the dependent variable (i.e., suicide attempts) in the logistic regression analysis in Step 1. In Step 2, simple linear regression analysis was conducted in order to confirm a relationship between the independent variables and the mediator variable (i.e., depression). In Step 3, logistic regression analysis was used to confirm relationships between the mediator and the dependent variable after controlling for the predictor variables. Then, we confirmed the relationships between the independent variables and the dependent variable after controlling for the mediator in Step 4. The Sobel test (58) was then employed in order to determine the indirect effects of the mediator variable on the association between predictor variables and the outcome variable. All analyses were conducted with SPSS version 21.0 for Windows (SPSS, Inc., and IBM Company, Chicago, IL, USA).

## Results

### Demographic Characteristics, Related Risk, and Positive Factors With Suicide Attempts

Sociodemographic and psychiatric characteristics, childhood trauma history, and positive psychological factors of subjects in the two groups (having a history of suicide attempts and having no history of suicide attempts) are shown in Table 1. Approximately two-thirds (75.2%) of participants were classified with no suicide attempt history while approximately one-third (24.8%) of participants were classified with suicide attempt history. Five hundred and fifty-four participants without suicide attempt history had a mean age of 38.3 (±13.4) years with 305 females (55.5%) and 245 males (44.5%). The mean years of education was 14.3(±2.8) years. Half of them were unmarried (50.2%) and majority of them were employed (56.0%). On the other hand, 183 participants with a history of suicide attempts had a mean age of 30.7 (±11.1) years with 97 females (53.3%) and 85 males (46.7%). The mean years of education was 13.6 (±2.4) years. Majority of them were unmarried (76.5%) and unemployed (59%). Comparing sociodemographic factors between the two groups according to suicide attempt history, subjects with a history of suicide attempts tended to be younger (*p* < 0.001), less educated (*p* = 0.002), be less employed (*p* = 0.001), and live alone (*p* < 0.001). There were no significant gender differences between the two groups.

**Table 1:** Demographic and psychiatric characteristics, childhood trauma, and positive factors of subjects with anxiety and/or depression by suicide attempt history.

**Variable**	**No suicide attempt history**	**Suicide attempt history**	***p^a^***
	**(*n =* 554, 75.2%)**	**(*n =* 183, 24.8%)**	
**DEMOGRAPHIC CHARACTERISTICS**			
Age, years, mean ± SD	38.3 ± 13.4	30.7 ± 11.1	<0.001^**^
Gender (female), no. (%)	305 (55.5)	97 (53.3)	0.668
Education, years, mean ± SD	14.3 ± 2.8	13.6 ± 2.4	0.002^**^
Marriage status (Unmarried), no. (%)	278 (50.2)	119 (76.5)	<0.001^**^
Employment status (no), no. (%)	244 (44.0)	108 (59.0)	0.001^**^
**PSYCHIATRIC CHARACTERISTICS**			
Depression (BDI), mean ± SD	21.8 ± 10.9	31.0 ± 10.7	<0.001^**^
Anxiety (STAI), mean ± SD	115.4 ± 23.9	130.4 ± 19.6	<0.001^**^
**CHILDHOOD TRAUMA (CTQ)**			
Emotional abuse (yes), no. (%)	8.2 (4.5)	11.6 (5.8)	<0.001^**^
Physical abuse (yes), no. (%)	8.6 (4.5)	10.8 (5.5)	<0.001^**^
Sexual abuse (yes), no. (%)	6.1 (2.6)	7.5 (4.3)	<0.001^**^
Emotional neglect (yes), no. (%)	12.5 (5.4)	15.1 (5.9)	<0.001^**^
Physical neglect (yes), no. (%)	8.0 (3.3)	8.7 (3.6)	0.035^*^
**POSITIVE FACTORS**			
Spirituality (FACIT-Sp-12), mean ± SD	22.1 ± 10.0	13.7 ± 9.4	<0.001^**^
Social support (FSSQ), mean ± SD	40.3 ± 11.6	35.2 ± 12.0	<0.001^**^

*BDI, Beck Depression Inventory; STAI, State-Trait Anxiety Inventory; CTQ, Childhood Trauma Questionnaire; FACIT-Sp-12, Functional Assessment of Chronic Illness Therapy-Spiritual Well-being Scale; FSSQ, Functional Social Support Questionnaire; SD, standard deviation*.

a *^a^Statistical significance from independent t-tests or chi-square tests*.

* p < 0.05,

** *p < 0.01*.

The mean BDI and STAI scores of participants with a history of suicide attempts were 31.0 (±10.7) and 130.4 (±19.6) which belong to the ranges of severe depression and anxiety (45, 59–61). These scores were significantly higher than the scores of participants with no history of suicide attempts (*p* < 0.001). People with a history of suicide attempts had significantly higher scores in all areas of childhood trauma questionnaires than those without a history of suicide attempts (*p* < 0.001). Considering positive factors, subjects with suicide attempt history tended to have lower FACIT-Sp-12 (*p* < 0.001) and FSSQ (*p* < 0.001) scores than those without suicide attempt history. In other words, participants with a history of suicide attempts had lower spirituality and social support than those with no history of suicide attempts.

### Independent Effect of Negative and Positive Factors on Suicide Attempts

Table 2 shows the results of a hierarchical multivariate logistic regression analysis examining the factors related to suicide attempts. After controlling for demographic covariates, a high grade of childhood trauma, low spirituality, low social support, and high levels of depression and anxiety symptoms were included in the model step by step. In the final model, being of a young age [adjusted [adj.] OR = 0.97 (0.95–1.00)] and having experienced a high level of emotional abuse [adj. OR = 1.06 (1.01–1.12)] and a high level of sexual abuse [adj. OR = 1.09 (1.02–1.17)], as well as low spirituality [adj. OR = 0.94 (0.91–0.98)] were all independently associated with suicide attempts. In particular, a high grade of childhood sexual abuse was the most influential variable on suicide attempts. The overall model fits the data well (Hosmer and Lemeshow test of goodness-of-fit χ^2^ = 8.08, *p* = 0.426).

**Table 2:** Results of hierarchical logistic regression model to identify factors related to suicide attempts.

**Block no., variable**	**Results of each step using forward**						**Last step results**	
	**stepwise method in blocks 2–4**							
	**OR (95%CI)**	***p***	**OR (95%CI)**	***p***	**OR (95%CI)**	***p***	**OR (95%CI)**	***p***
**CONSTANT**								
1. Covariates								
Age, years	0.97 (0.95–0.99)	0.001^**^	0.97 (0.95–0.99)	0.001^**^	0.98 (0.96–0.10)	0.027^*^	0.97 (0.95–1.00)	0.017^*^
Gender (female)	0.76 (0.52–1.12)	0.164	0.90 (0.60–1.35)	0.625	0.89 (0.58–1.35)	0.571	0.98 (0.64–1.50)	0.914
Marriage status (Unmarried)	0.44 (0.26–0.74)	0.002^**^	0.53(0.31–0.91)	0.022^*^	0.68 (0.39–1.19)	0.176	0.65 (0.37–1.14)	0.135
Education, years	0.96 (0.89–1.03)	0.208	0.97 (0.90–1.05)	0.476	0.98 (0.90–1.06)	0.556	0.98 (0.91–1.07)	0.680
Employment status (no)	0.68 (0.47–0.99)	0.043^*^	0.71 (0.48–1.05)	0.085	0.76 (0.50–1.14)	0.182	0.78 (0.51–1.18)	0.239
2. Childhood trauma								
Emotional abuse (yes)			1.07 (1.02–1.12)	0.011^*^	1.07 (1.02–1.13)	0.010^*^	1.06 (1.01–1.12)	0.028^*^
Physical abuse (yes)			1.02 (0.97–1.07)	0.465	1.02 (0.97–1.07)	0.497	1.01 (0.96–1.06)	0.611
Sexual abuse (yes)			1.09 (1.03–1.16)	0.005^**^	1.09 (1.03–1.17)	0.007^**^	1.09 (1.02–1.17)	0.012^*^
Emotional neglect (yes)			1.03 (0.98–1.07)	0.242	1.00 (0.96–1.05)	0.966	1.01 (0.96–1.06)	0.665
Physical neglect (yes)			0.98 (0.92–1.05)	0.616	0.99 (0.92–1.06)	0.737	0.98 (0.91–1.06)	0.628
3. Positive factors								
Spirituality (FACIT–Sp-12)					0.93 (0.90–0.96)	<0.001^**^	0.94 (0.91–0.98)	0.001^**^
Social support (FSSQ)					1.01 (0.99–1.03)	0.562	1.01 (0.99–1.03)	0.344
4. Psychiatric characteristics								
Depression (BDI)							1.05 (1.02–1.08)	0.001^**^
Anxiety (STAI)							0.10 (0.98–1.01)	0.693
**MODEL FIT**								
Hosmer and Lemeshow test	χ^2^ = 5.694, *p* = 0.681		χ^2^ = 1.842, *p* = 0.985		χ^2^ = 2.223, *p* = 0.973		χ^2^ = 8.080, *p* = 0.426	

*BDI, Beck Depression Inventory; STAI, State–Trait Anxiety Inventory; CTQ, Childhood Trauma Questionnaire; FACIT-Sp-12, Functional Assessment of Chronic Illness Therapy-Spiritual Well-being Scale; FSSQ, Functional Social Support Questionnaire*.

*Hierarchical multivariate logistic regression analysis was performed*.

* p < 0.05,

** *p < 0.01*.

### Mediating Effects of Depression on Patients' Suicide Attempts

**Figure 1:**
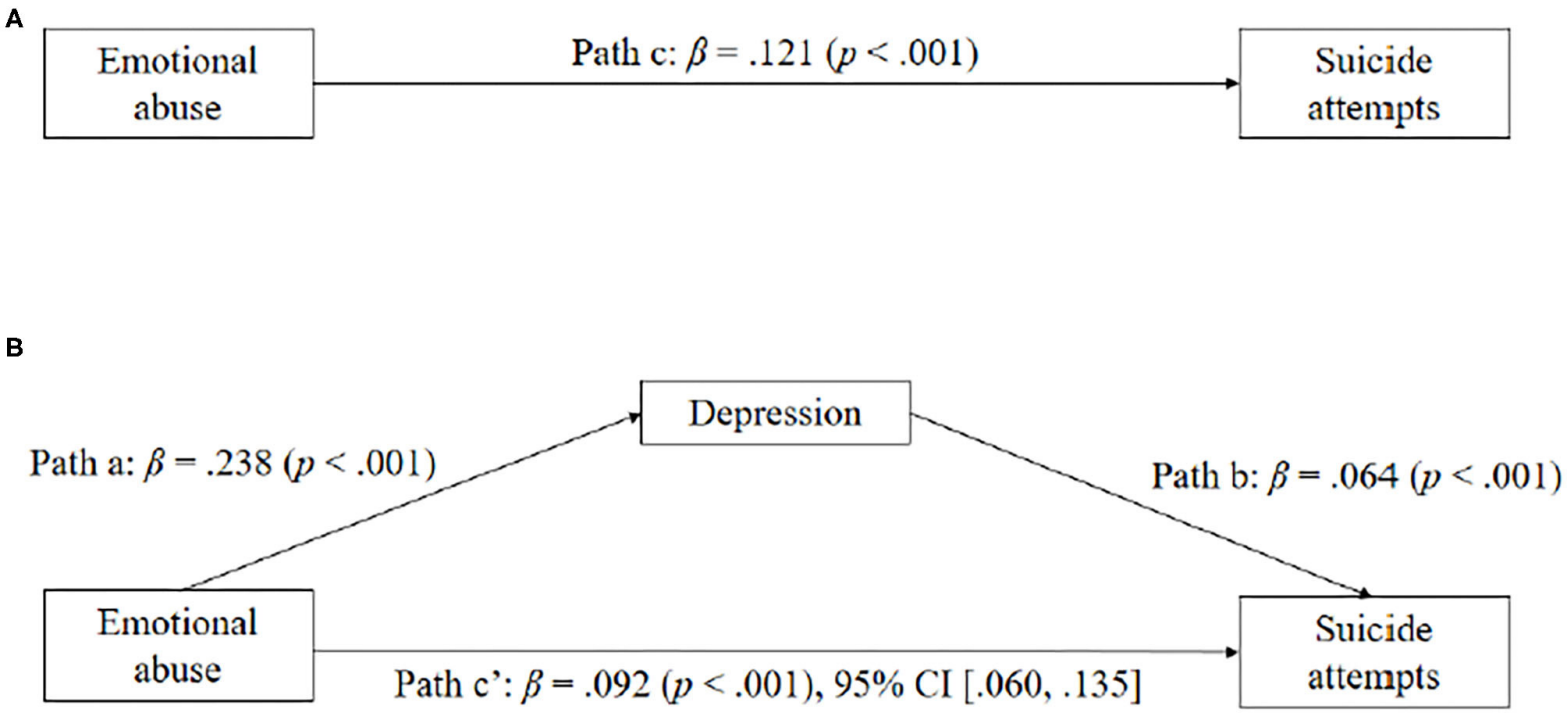
Depressive symptoms as mediators between childhood emotional abuse and suicide attempts. Standardized coefficients are shown for each path (with *p* in parentheses).

All four steps of Baron and Kenny's analysis were performed in order to examine the mediating roles of depressive symptoms in the relationships between childhood emotional abuse, childhood sexual abuse, spirituality, and suicide attempts. As shown in Table 3, the results of mediation analyses showed that the total effect of childhood emotional abuse on suicide attempts was significant (β = 0.121, SE = 0.016, *p* < 0.001). The significant coefficient of path a (β = 0.238, SE = 0.988, *p* < 0.001) and path b (β = 0.064, SE = 0.009, *p* < 0.001) indicated positive associations of childhood emotional abuse on depression, and depression on suicide attempts. Besides, the indirect effect of childhood emotional abuse on suicide attempts through depression was statistically significant (β = 0.092, SE = 0.017, *p* < 0.001). Therefore, depression significantly mediated the effect of childhood emotional abuse on suicide attempts (see Figure 1).

**Table 3:** Mediating effects of depression on patients' suicide attempts.

	**β**	***SE***	***p***	**Exp(B)**	**Adjusted *R^**2**^***
Model 1					
Emotional abuse → Suicidal attempt	0.121	0.016	<0.001	1.129	0.110
Emotional abuse → Depression	0.238	0.988	<0.001		0.055
Depression → Suicidal attempt	0.064	0.009	<0.001	1.066	0.216
Emotional abuse → Suicidal attempt excluding Depression	0.092	0.017	<0.001	1.097	
Sobel test *z* = −4.36, *p* = <0.001					
Model 2					
Sexual abuse → Suicidal attempt	0.116	0.025	<0.001	1.123	0.044
Sexual abuse → Depression	0.138	1.092	<0.001		0.018
Depression → Suicidal attempt	0.069	0.008	<0.001	1.072	0.186
Sexual abuse → Suicidal attempt excluding Depression	0.087	0.027	0.001	1.091	
Sobel test *z* = −2.35, *p* = 0.001					
Model 3					
Spirituality → Suicidal attempt	−0.094	0.011	<0.001	0.910	0.187
Spirituality → Depression	−0.492	0.740	<0.001		0.241
Depression → Suicidal attempt	0.044	0.010	<0.001	1.045	0.220
Spirituality → Suicidal attempt excluding Depression	−0.065	0.012	<0.001	0.938	
Sobel test *z* = −3.31, *p* = <0.001					

*Baron and Kenny's procedures were used to examine the mediating effects of depression*.

**Figure 2:**
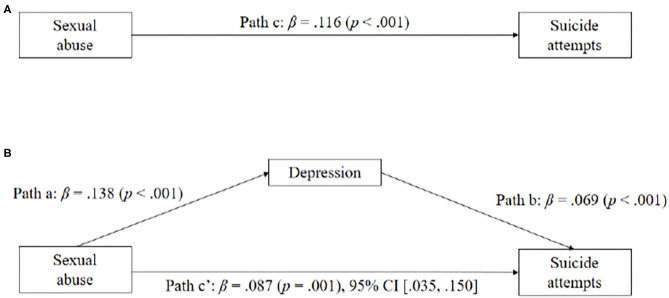
**(A,B)** Depressive symptoms as mediators between childhood sexual abuse and suicide attempts. Standardized coefficients are shown for each path (with p in parentheses).

Significant effects were also observed for childhood sexual abuse on depression (β = 0.138, SE = 1.092, *p* < 0.001), depression on suicide attempts (β = 0.069, SE = 0.008, *p* < 0.001), and childhood sexual abuse on suicide attempts (β = 0.116, SE = 0.025, *p* < 0.001). Notably, the indirect effect of childhood sexual abuse on suicide attempts through depression was significant (β = 0.087, SE = 0.027, *p* = 0.001). The pattern of the mediating effects of depression between childhood sexual abuse and suicide attempts is depicted in Figure 2.

**Figure 3:**
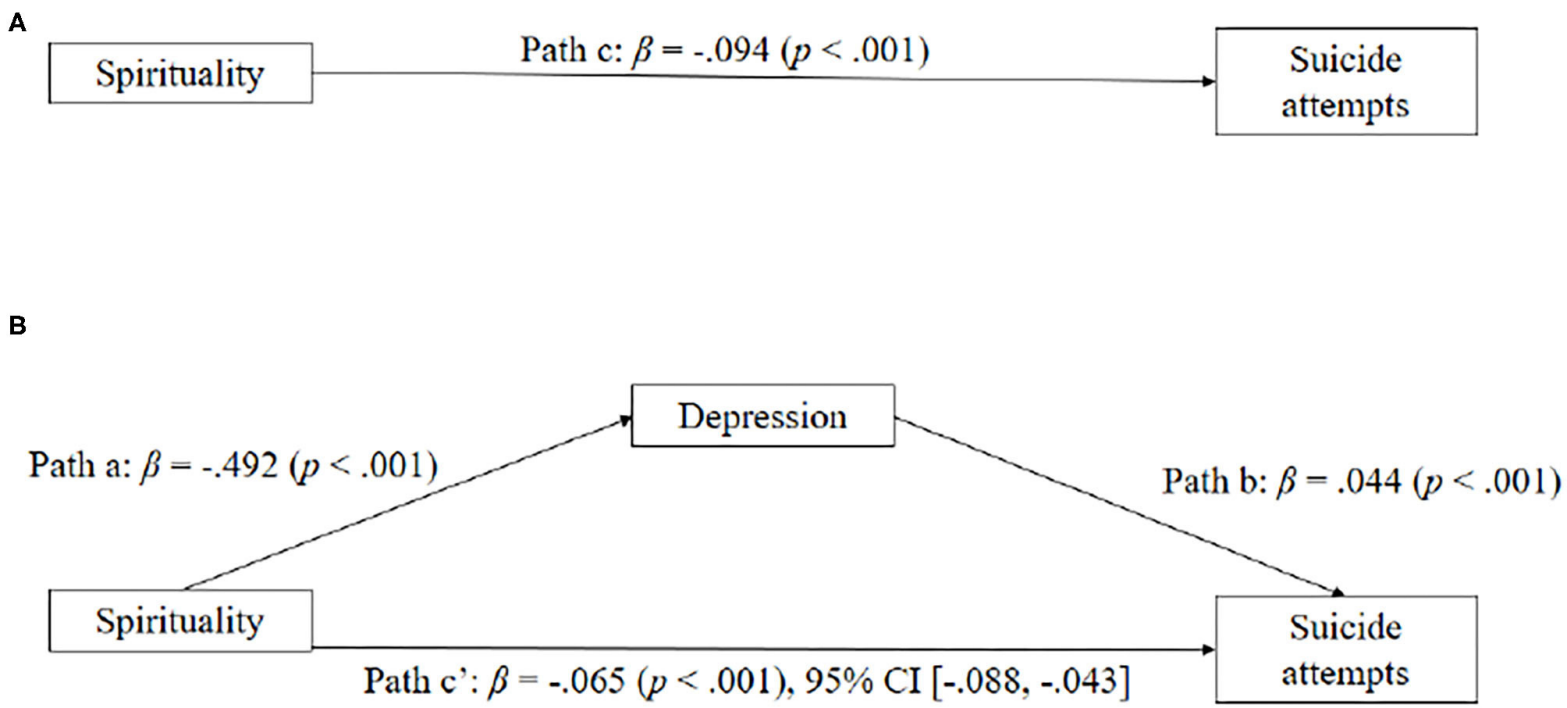
**(A,B)** Depressive symptoms as mediators between spirituality and suicide attempts. Standardized coefficients are shown for each path (with *p* in parentheses).

Figure 3 displays the model of spirituality as a predictor of suicide attempts, mediated by depression. When depression was excluded in the model, spirituality significantly predicted suicide attempts (β = −0.094, SE = 0.011, *p* < 0.001). The simple mediation analysis with suicide attempts as the outcome indicated that spirituality significantly predicted depression (β = −0.492, SE = 0.740, *p* < 0.001). The association was negative: as spirituality increased, depression severity declined (and vice versa). Spirituality significantly predicted suicide attempts even with relationship depression in the model (β = −0.065, SE = 0.012, *p* < 0.001) and depression also significantly predicted suicide attempts (β = 0.044, SE = 0.010, *p* < 0.001). As depression severity increased, suicide attempts increased as well (and vice versa), and as spirituality increased, suicide attempts decreased. Consistent with our hypothesis, depression significantly mediated the effect of spirituality on suicide attempts.

## Discussion

In the present study on patients with depression and/or anxiety disorders, being young, having experienced a high level of childhood emotional and sexual abuse, and having a high level of depression and low level of spirituality were all significant independent factors for increased suicide attempts after controlling for sociodemographic factors, childhood trauma history, positive psychological factors, and psychiatric symptoms. In particular, depressive symptoms were identified as partially mediating the relationships between childhood emotional and sexual abuse, spirituality, and suicide attempts.

The findings of our study were consistent with those of previous studies that depression was significantly related to suicidality (62–64). It is therefore important to identify the close relationship between suicide and depression distinct from anxiety. The first possible connecting link between these two factors is hopelessness. Wolfe et al. (65) reported that hopelessness is a mediator of suicidal ideation in depressive adolescent youth. A strong relationship among depression, suicide, and hopelessness as a mediator was demonstrated in a non-clinical sample in another study (66). The second factor affecting suicidality was identified as anhedonia. Research has suggested that anhedonia may be a unique symptom of depression associated with thoughts of suicide (63). Those who have suicidality are less interested in experiencing pleasure and try to avoid intolerable psychological pain, which motivates them to think about suicide (64). In other words, hopelessness and anhedonia, which are intimately related with depression other than anxiety, are important factors associated with suicide.

Several studies have reported that sexual abuse history in particular has a noticeable effect on the risk of suicide (67–69). Brown et al. proposed that, among various types of childhood trauma, sexual abuse alone was the strongest independent risk factor for suicide (70). Ryan demonstrated that those who had a history of childhood sexual trauma may respond to anger, even if there is no danger related to the traumatic experience (71). The effects of traumatic events and depression on anger, particularly internal hostility, are related to suicide risk, suggesting a potential mechanism of childhood sexual trauma and suicide linkage (72). On the other hand, childhood emotional abuse is an independent risk factor of suicide with its effects on interpersonal relationships (73). Emotional difficulties arousing from negative relationships between the exploiter caregiver and the abused child may be internalized and responsible for long-term effects on negative cognitions (74). Those with a history of emotional abuse may have schemas of deficiency, shame, and self-sacrifice, which may induce emotional problems and depression (75). Therefore, it was concluded that sexual and emotional abuse was a significant determinant of predisposition to suicide.

As our study has shown, spirituality was a significant factor having a meaningful protective effect on suicide. It has been reported that spirituality may increase senses of intimacy as well as enhance a feeling of comfort and relief (76), provide hope in one's life (77, 78), and decrease physical, psychological, and social difficulties (76, 79, 80). High spirituality helps individuals apply a positive coping style against stress (81) and decrease suicide in various populations (82–84). Wu et al. (85) has shown that religion is a positive factor against completed suicide in a majority of settings where suicide research is conducted using a meta-analysis. Thus, taking a spiritual history is not only necessary to identify spiritual resources that can be used to facilitate psychological well-being, but also to identify ability that may directly impact on suicide prevention.

A spiritual history can be included as part of the social history at the time of hospital admission, during a new patient evaluation, or as part of an outpatient visit (86). There is also evidence that addressing spiritual issues enhances the doctor-patient relationship and helps to build trust (87). If spiritual needs are identified, then spirituality-based intervention can be introduced. Especially, mindfulness is a practice that has long been associated with spiritual development (88, 89). Because mindfulness has its roots in Asian culture, it especially can be an effective intervention to enhance spirituality among Koreans. Yong et al. demonstrated that spirituality training program including mindfulness meditation showed beneficial effects on spiritual well-being for middle manager nurses in Korea (90). The significant improvement in spiritual well-being and spiritual integrity in the experimental group was supported by similar results in other health care professionals (91, 92). Therefore, spirituality-based programs can be introduced within continuing education and staff development programs for mental health professionals.

Although there is some evidence that social support is associated with decreasing suicide risk in patients with depression (93, 94), we did not identify this independent relationship among the two. Social support is defined as the perception of the individual regarding relationships with other positive resources that assist the individuals to cope not only with every day events, but also with stressful situations (95, 96). Because FSSQ aims to measure the person's satisfaction with functional and affective aspects of the individual's social support (97, 98), not all aspects and sources of social support may be evaluated. Some studies have also suggested that depressive symptoms are mediating factors which affect the relationship between suicide and psychosocial factors (99, 100). Depression might weaken the protective effect of social support on suicide attempts. Therefore, the mediating effects of depressive symptoms on the association between social support and suicide should be considered in interpreting the results.

Our study does have some limitations. First, we used self-reporting scales to assess psychological symptoms, including BDI, STAI, CTQ, FACIT-Sp-12, and FSSQ; self-reporting can exaggerate the reported severity of symptoms (101, 102). Second, because of the cross-sectional design of this study, we cannot certainly infer causality between suicide attempts and the other variables studied. Therefore, further study with a prospective design which identifies causality may be needed in order to clarify how significant factors such as spirituality contribute to the alleviation of the effects of the risk factors on suicide. Third, despite the classification between anxiety and/or depressive disorder, we considered the two disorders as one group. One of the most prevalent findings in psychiatry is the frequent comorbidity between anxiety disorders and depressive disorders (103). Co-morbidity has many origins such as genetic factors (104, 105) and environmental experiences, including stressful life events (106, 107). Although anxiety disorders and depressive disorders have displayed frequent comorbidity, the specificity of each diagnosis may be identified in order to differentiate between the outcomes in the different populations (108). Fourth, despite the various severities of depressive symptoms, we regarded different depressive disorders as a single group. Because this study was performed at a general hospital located in Seoul, the capital of South Korea, participants coming all over the country had different associated symptoms and severities difficult to characterize a specific population. Also, participants had different comorbidities including physical and mental disorders (e.g., somatic symptom and related disorders, trauma- and stressor- related disorders, sleep-wake disorders etc.) Therefore, further study classifying depressive disorders with the severity and symptomatology may be required. Fifth, high risk groups are excluded from the study; exclusion criteria such as substance abuse could make a biased population due to the high comorbidity between substance use disorders and depressive/anxiety disorders. Sixth, our study could have been improved by using previous medical records. Because participants were joined and evaluated at their first visit, previous medical records of them could not be identified. We did not consider past substance abuse which could have contributed to suicidality. Finally, although suicide attempts are a definite behavioral indicator of suicide, they cannot evaluate the severity and frequency of suicidal ideation and any specific plans for suicide attempts. According to the continuum model of suicide, suicidal ideation has been shown to be an important indicator of future suicide attempts (109). Therefore, further study assessing various aspects of suicidality with several scales and detailed clinical interviews may be required.

Identifying independent factors associated with suicide attempts might be important for establishing effective plans of suicide prevention. Clinicians need to be aware of the types of childhood maltreatment and the broad range of household dysfunction they may encounter. An awareness of the relationship between specific types of childhood abuse and suicide attempts may benefit interventions for people with depression. In particular, early prevention efforts aimed at children who have experienced sexual and emotional abuse may reduce their risks for the development of suicide. In addition, an assessment of spirituality level in conjunction with psychiatric risk factors may also be recommended in screening patients at risk of suicide. A brief spiritual history is recommended for all patients visiting psychiatric clinics. The findings of this study highlight the associations between spiritual well-being and suicide in patients with depression, which should prompt clinicians to take into account spirituality in an effort to improve psychological well-being in patients with depression and/or anxiety. In conclusion, an assessment and strategic interventions to decrease depression and support spirituality might be significant for suicide prevention.

## Data Availability Statement

The raw data supporting the conclusions of this article will be made available by the authors, without undue reservation.

## Ethics Statement

The studies involving human participants were reviewed and approved by The Institutional Review Board of the Ethics Committee of Seoul St. Mary's Hospital at The Catholic University of Korea (KC09FZZZ0211). The patients/participants provided their written informed consent to participate in this study.

## Author Contributions

HT was involved in conceiving of the study, carrying out all aspects of the data collection and analysis, and writing of the manuscript. J-HC provided critical feedback and aided in editing the first draft. All authors contributed to and have approved the final manuscript.

## Conflict of Interest

The authors declare that the research was conducted in the absence of any commercial or financial relationships that could be construed as a potential conflict of interest.
